# Diarrhoea in a large prospective cohort of European travellers to resource-limited destinations

**DOI:** 10.1186/1471-2334-10-231

**Published:** 2010-08-04

**Authors:** Raffaela Pitzurra, Robert Steffen, Alois Tschopp, Margot Mutsch

**Affiliations:** 1 University of Zurich, Institute for Social and Preventive Medicine, Division of Epidemiology and Prevention of Communicable Diseases and World Health Organization Collaborating Centre for Travellers' Health, Zurich, Switzerland; 2 University of Zurich, Institute for Social and Preventive Medicine, Biostatistics Division, Zurich, Switzerland

## Abstract

**Background:**

Incidence rates of travellers' diarrhoea (TD) need to be updated and risk factors are insufficiently known.

**Methods:**

Between July 2006 and January 2008 adult customers of our Centre for Travel Health travelling to a resource-limited country for the duration of 1 to 8 weeks were invited to participate in a prospective cohort study. They received one questionnaire pre-travel and a second one immediately post-travel. First two-week incidence rates were calculated for TD episodes and a risk assessment was made including demographic and travel-related variables, medical history and behavioural factors.

**Results:**

Among the 3100 persons recruited, 2800 could be investigated, resulting in a participation rate of 89.2%. The first two-weeks incidence for classic TD was 26.2% (95%CI 24.5-27.8). The highest rates were found for Central Africa (29.6%, 95% CI 12.4-46.8), the Indian subcontinent (26.3%, 95%CI 2.3-30.2) and West Africa (21.5%, 95%CI 14.9-28.1). Median TD duration was 2 days (range 1-90). The majority treated TD with loperamide (57.6%), while a small proportion used probiotics (23.0%) and antibiotics (6.8%). Multiple logistic regression analysis on any TD to determine risk factors showed that a resolved diarrhoeal episode experienced in the 4 months pre-travel (OR 2.03, 95%CI 1.59-2.54), antidepressive comedication (OR 2.11, 95%CI 1.17-3.80), allergic asthma (OR 1.67, 95%CI 1.10-2.54), and reporting TD-independent fever (OR 6.56, 95%CI 3.06-14.04) were the most prominent risk factors of TD.

**Conclusions:**

TD remains a frequent travel disease, but there is a decreasing trend in the incidence rate. Patients with a history of allergic asthma, pre-travel diarrhoea, or of TD-independent fever were more likely to develop TD while abroad.

## Background

Travellers' diarrhoea (TD), the most common health problem in visitors to tropical and subtropical destinations, affects between 20 to over 60% of persons during a two weeks stay in a high risk country such as India or Kenya [1-3]. As many of those data have been generated some decades ago, the aim of this study is to provide updated region-based TD incidence rates, and to investigate risk factors.

## Methods

### Study design

Based on a protocol approved by the Ethical Commission of the Canton of Zurich, we performed a prospective questionnaire-based cohort study.

### Study population

Potential participants were recruited at the Centre for Travel Health of the University of Zurich between July 2006 and January 2008. To be included, participants had to be adult, German-speaking Swiss residents who planned to travel to high-risk TD destinations [2,4] for a duration of 1 to 8 weeks. Subjects planning to take prophylactic antibiotics during their trip, those who reported a history of severe illness (anaemia, cancer, immunodeficiency or immunosuppressive disorders, severe psychiatric illness, previous gastrointestinal surgery), with functional organic gastrointestinal disorders (according to Rome II [5]/Rome III [6] criteria), or recurring diarrhoeal symptoms within a four months pre-travel period, and pregnant women were excluded.

### Definitions

Classic TD was defined as three or more unformed stools per 24 h with at least one accompanying symptom (nausea, vomiting, abdominal cramps, tenesmus, fever, blood in stools)[7,8], while 3 or more unformed stools with or without additional symptom(s) was named TD based on the UNICEF/WHO definition [9,10]. Patients experiencing one or two loose stools per 24 h while abroad were classified as having mild diarrhoea [8,10]. TD and classic TD were used for incidence calculations in the first two weeks of stay abroad and only TD for multiple logistic regression analysis. TD patients, who reported fever or blood in their stools were rated as having dysentery. Multiple episodes of TD had to be separated by a TD-free interval of at least 72 hours. Incapacitation meant an inability to pursue planned travel activities and was rated in 3 subgroups; low incapacitation could pursue all activities, and severe was defined by being confined to bed for at least 12 hours or by consulting a doctor [4,7]. Body Mass Index (BMI) was calculated from participants' height and weight data at enrolment. Origin was the country in which the participant spent the first five years of life. 'Newcomers' were visiting the index travel region for the first time. Countries and subcontinents were grouped according to the United Nations World Migrant Stock [11]. Comedication and diseases were classified according to the main categories of the International Classification of Diseases (ICD-10 2007)[12]. Allergies formed a separate disease entity including allergic asthma, allergic rhinitis, hymenoptera and atopic dermatitis, those were self-reported by the study participant, but an MD confirmation or diagnosis was requested.

### Study conduct

Participants who visited our Centre for Travel Health for standard pre-travel consultation were invited to participate in the study on a voluntary basis. Pre-travel advice regarding TD included an explanation of TD etiology, mode of pathogen transmission, preventive behaviour, treatment options and, if indicated, written instructions on how to use antibiotics. A leaflet on general medications (including such against TD) was provided as part of a travel medical kit. Upon signing an informed consent, the participants received two questionnaires. Q1 was collected immediately upon completion, while Q2 was to be returned in the first week after their return reminded either by mail or email; Q2 was similar to a diary.

Q1 included 30-structured questions to assess the itinerary, previous travel to the tropics, demographic data, body mass index, chronic diseases, confirmed allergies, and pre-travel diarrhoea characteristics. In addition, adverse life events in the preceding 12 months, self-reported stress, smoking habits and alcohol consumption, and perceived susceptibility to diarrhoea were investigated.

Q2 consisted of 17-questions to confirm the travel itinerary and to document a detailed history of TD abroad, including TD medication used. After an initial trial phase of three months we added questions about syndromes abroad (health impairments reported abroad, i.e. TD independent fever) and attitudes towards diarrhoea (catering, adherence to 'cook it, boil it, peel it or forget it', tap water consumption) [13,14]. Non-responders were reminded by mail twice; patients reporting uncured diarrhoea were followed until resolution. Those who refused to respond to Q2 were interviewed with the single question whether they had experienced diarrhoea while abroad. No stool samples were collected in this study.

### Statistical analysis

Data were analysed using Stata statistical software, version 10.1 (Stata).

The 95% confidence intervals (CI) for the incidence rates in various subcontinents were estimated as per Newcombes' exact (not approximative) method [15]. We compared differences in proportions of demographics, attitudes and clinical variables using the chi square tests. The significance level was set at α (alpha) = 0.05. All travel- and traveller-related risk factors were evaluated as independent potential risk factors for the development of TD in a multiple logistic regression model. Odds ratios (OR) were determined by stepwise backward elimination of variables with *p *> 0.100. For the multiple logistic regression analysis only, the later inserted question referring to TD-independent fever was analysed by imputing with organising the missing data using the pattern of gender, destination continent and education. Single imputation by regression imputes for cases with missing values on a variable instead of mean values of this variable function values for this variable out of a regression equation with the following independent variables: education, gender and destination. For the sensitivity tests, the results of the multiple analysis of the complete dataset were compared first to a selection of half of the data and second, to the classic TD definition.

## Results

### Demographics

**Figure 1:**
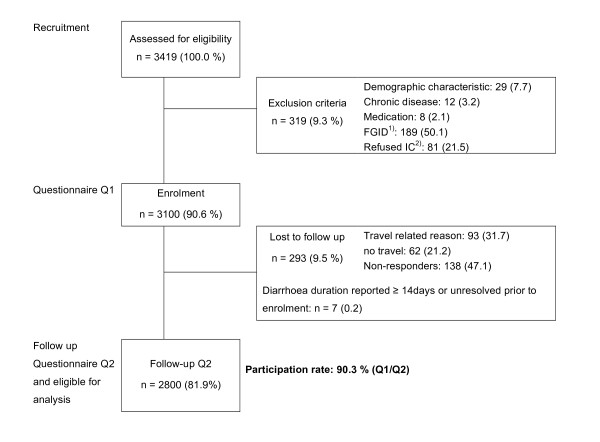
**Overview of prospective participants recruitment**. ^1) ^functional gastrointestinal disease. ^2) ^Informed consent

**Figure 2:**
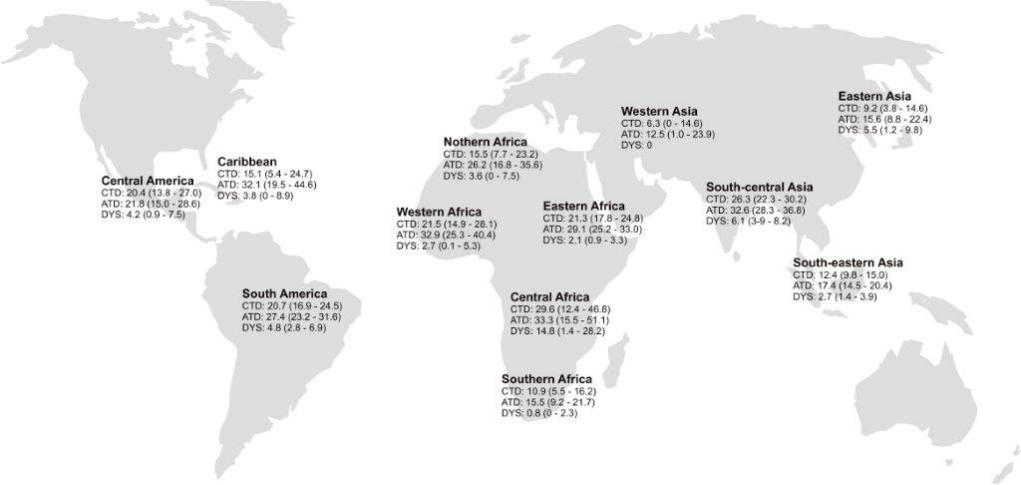
**First two-week-incidences in selected areas**. (N = 2794). CTD classic TD (n = 523). ATD all TD (n = 710). DYS dysentery (n = 166)

Among 3100 enrolled travellers, 2800 (90.3%) were eligible for analysis (figure 1). Gender was approximately equally distributed (Table 1). Mean age was 38.8 ± 12.6 years (median 35) and half of the population (1403, 51.1%) had a university degree, with significant higher proportions among the business travellers (*p *= 0.0057). Mean travel duration was 3.2 weeks with the most frequently visited destinations being Southeast (SE) Asia (636, 22.7%), followed by East Africa (522, 18.6%), the Indian subcontinent (476, 17.0%) and South America (435,15.5%). The classic TD and all TD incidence rates for various regions are shown in figure 2 with the highest rates reported in most parts of Africa and on the Indian subcontinent. The largest group of travellers were tourists (1539; 87.9%), while 121 (10.8%) went for business; 90 (5.1%) visited friends and relatives (VFR). The median travel time for business was 2 weeks, while VFR and tourists travelled for a median duration of 3 weeks. A significantly higher proportion of businessmen visited the Indian subcontinent (44; 15.3%, *p *< 0.0001) and was male (69; 57.0%, *p *= 0.0132). Africa was the preferred continent of tourists (508, 33.1%), while VFR mainly travelled to Latin America (38; 42.2%) and Asia (22; 24.4%). A minority visited the tropics or subtropics for the first time (208, 7.5%), among whom 92 (44.2%) rated themselves as intermediate to very susceptible to diarrhoea, compared to 940 (36.7%) among experienced travellers. Post-travel questionnaires were returned within a median of 10 days (interquartile range 2-30) after returning home.

**Table 1:** Demographic and selected behavioural factors of the travellers' cohort.

	Total		subgroups		
		TD n = 962	mild diarrhoea n = 303	no diarrhoea abroad n = 1535	Classic TD n = 785
Sex	n = 2800				
Female	1406 (50.2)	466 (33.1)	151 (10.7)	789 (56.1)	400 (28.4)
Male	1394 (49.8)	496 (35.6)	152 (10.9)	746 (53.5)	385 (27.6)
Age group, years ^1)^	n = 2672				
18-30	829 (31.0)	338 (40.8)	93 (11.2)	389 (46.9)	292 (35.2)
31-40	882 (33.0)	283 (32.1)	102 (11.6)	497 (56.3)	230 (26.1)
41-60	734 (27.5)	242 (33.0)	76 (10.4)	416 (56.7)	186 (25.3)
>60	227 (8.5)	55 (24.2)	18 (7.9)	154 (67.8)	40 (17.6)
Origin (first 5 years of life)	n = 2764				
European	2616 (94.6)	899 (34.4)	287 (11.0)	1430 (54.7)	734 (28.0)
TD high risk country^2)^	75 (2.7)	25 (33.3)	6 (8.0)	44 (58.7)	19 (25.3)
other	73 (2.6)	24 (32.9)	8 (10.9)	41 (56.2)	19 (26.0)
Education	n = 2745				
Vocational school level 1 ^3)^	588 (21.4)	196 (33.3)	71 (12.1)	324 (55.1)	151 (25.7)
Vocational school level 2 ^4)^	754 (27.5)	258 (34.2)	75 (9.9)	421 (55.8)	212 (28.1)
University	1403 (51.1)	504 (35.9)	152 (10.8)	758 (54.0)	407 (29.0)
Destination^5)^	n = 2790				
Africa	903 (32.4)	313 (34.7)	107 (11.8)	483 (53.5)	246 (27.2)
Asia	1266 (45.4)	407 (32.1)	126 (10.0)	733 (57.9)	335 (26.5)
Latin America	617 (22.1)	234 (37.9)	70 (11.3)	313 (50.7)	202 (32.7)
Travel type	n = 1750				
Tourism	1539 (87.9)	545 (35.4)	173 (11.2)	821 (53.3)	444 (28.8)
Family/VFR	90 (5.1)	42 (46.6)	15 (16.6)	33 (36.7)	36 (40.0)
Business	121 (10.8)	37 (30.6)	10 (8.3)	74 (61.2)	27 (22.3)
Travel duration, weeks ^1)^	n = 2800				
mean ± sd	3.2 ± 1.6	3.6 ± 1.9	3.2 ± 1.6	3.0 ± 1.4	3.7 ± 1.9
median (range)	3 (1-8)	3 (1-8)	3 (1-8)	3 (1-8)	3 (1-8)
1-3 weeks	1849 (66.0)	560 (30.3)	206 (11.1)	1083 (58.6)	447 (24.2)
3.5 -5 weeks	674 (24.1)	249 (36.9)	74 (11.0)	351 (52.1)	205 (82.3)
5.5-8 weeks	277 (9.9)	153 (55.2)	23 (8.3)	101 (36.5)	133 (48.0)
History of travel	n = 2768				
Experienced	2560 (92.5)	867 (33.9)	275 (10.7)	1418 (55.4)	706 (27.6)
Newcomer (first such journey)	208 (7.5)	84 (40.4)	24 (11.5)	100 (48.1)	70 (33.6)
Smoking habits					
	n = 2788				
previous smoker	1041 (37.3)	369 (35.4)	100 (9.6)	572 (54.9)	299 (28.7)
previous non-smoker	1747 (62.7)	590 (33.7)	201 (11.5)	956 (54.7)	483 (27.6)
	n = 2793				
smoker	872 (31.2)	314 (36.0)	81 (9.3)	477 (54.7)	263 (30.2)
non-smoker	1921 (68.8)	644 (33.5)	221 (11.5)	477 (24.8)	519 (27.0)
Daily alcohol consumption	n = 2790				
yes	594 (21.3)	200 (33.7)	70 (11.8)	324 (54.5)	161 (27.1)
>1 glass daily	90 (15.4)	32 (35.6)	10 (11.1)	48 (53.3)	24 (26.7)
no	2196 (78.7)	757 (34.5)	231 (10.5)	324 (14.7)	620 (28.2)

**NOTE**. TD is travellers' diarrhoea, three or more unformed stools with or without additional symptom, classic TD with at least one accompanying symptom, mild diarrhoea one or two unformed stools per 24 hours. VFR is visiting friends and relatives.

^1) ^Significant. Chi square test, *p *< 0.05 was considered statistically significant. TD patients were compared to those with no diarrhoea abroad. All other factors not significant.

^2) ^Defined by Steffen et al. [2,4]

^3) ^Swiss obligatory school to apprenticeship school

^4) ^Swiss *Matura *(baccalaureat, general qualification for university access) and federal diplomas

^5) ^Oceania was omitted as only 4 travellers had visited this region

Among other diseases (596; 25.8%), common cold (277, 9.9%) and headache (106; 3.8%) were most reported. Fever independent from TD was mentioned by 45 (2.5%) study participants.

### Travellers' diarrhoea

**Figure 3:**
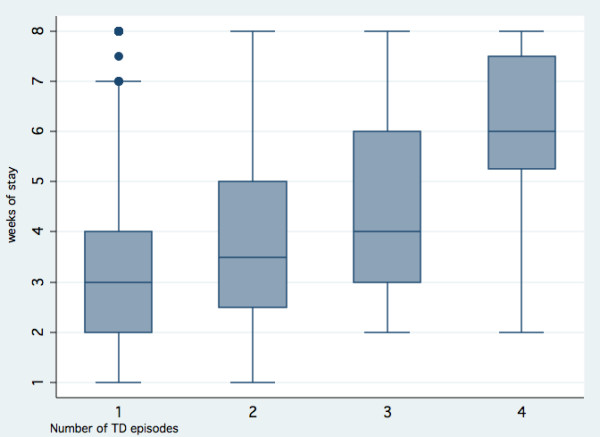
**Number of TD episodes by weeks of stay within a high-risk region**. (n = 821). High-risk TD regions as defined by Steffen et al. [2,4]. 1) 1 episode (n = 573). Median 3 (1-8), mean 3.4 ± 1.7 weeks. 2) 2 episodes (≥72 h-interval) (n = 189). Median 3.5 (1-8), mean 4.1 ± 2.0 weeks. 3) 3 episodes (≥72 h-interval) (n = 51). Median 4 (2-8), mean 4.4 ± 2.0 weeks. 4) 4 episodes (≥72 h-interval) (n = 8). Median 6 (2-8), mean 5.9 ± 2.0 weeks. Participants with missing episodes n = 141 (14.7%)

Among the 2800 exposed, 962 had TD, resulting in a TD attack rate of 34.4% (95% CI 32.6-36.1) and in a worldwide TD incidence for the first two-weeks stay of 26.2% (95% CI 24.5-27.8). A majority of 573 volunteers (69.8%) reported a single TD episode, 248 (30.2%) suffered from more than one episode while abroad. The number of TD episodes experienced increased approximately linearly with the duration of stay as illustrated in figure 3. A total of 785 suffered classic TD while abroad (28.0%; 95%CI 26.4-29.7) with a classic TD 2-weeks incidence of 19.9 (18.3-21.4 95%CI). A total of 303 (10.8%) experienced mild diarrhoea. The most frequent accompanying symptoms were tenesmus (56.0% among the TD patients) and cramps (49.2%). Fever (mean 38.4 ± 0.8°C) or vomiting was reported by approximately 15% (146 with fever, 136 vomiting), and 166 (17.3%) study subjects suffered from dysentery. The highest 2 weeks incidence rates for dysentery were recorded in Central and in Western Africa, and on the Indian subcontinent (figure 2). A total of 170 (17.7%) TD patients reported no accompanying symptom, but experienced a median of 4 stools per 24 hours (range 3-20).

The number of TD episodes reported had no significant influence on the use of antibiotics or probiotics (*p *= 0.282, *p *= 0.532, respectively). In contrast TD patients who reported treatment with loperamide (co-medication with antibiotics excluded) had more TD episodes (OR 1.45, 95% CI 1.05-2.19, *p *= 0.0026). TD occurred on average within 2.1 weeks after arrival (median 2 days) and patients counted on average 4 stools per day. One in every nine (111, 11.5%) experienced diarrhoea for longer than one week, with 37 (3.8%) individuals suffering from persistent (≥14 days of TD) and 11 (1.1%) from chronic diarrhoea (≥ 30 days). Roughly two thirds of patients (614, 63.8%) could pursue their planned activities, but 102 (10.8%) were confined to bed or consulted a physician, including 60 with dysentery (36.1% of all dysenteric patients).

Among treated patients, 343 (57.6%) chose an antimotility agent for self-medication of TD, 137 (23.0%) a probiotic and 51 combined both. A total of 116 (12.3%) TD patients reported intake of antibiotics, 65 of them (56.0%) for diarrhoea. The most frequently used antibiotics against TD were quinolones (e.g. ciprofloxacin), but trimethoprim-sulfamethoxazole were also reported (6 cases). The use of charcoal was reported by 70 persons (19.5%) and oral rehydration treatment was mentioned in 62 (6.4%) of the TD subjects and in 16 (9.6%) of dysenteric subjects. Among the dysentery cases, 35 (21.1%) were treated with an antibiotic, 79 (47.6%) used non-antibiotic medication exclusively, and this was most commonly loperamide (n = 38). Against advice, 152 (58.7%; *p *= 0.0191) travellers with an academic background reported consumption of tap water, and 60 (39.5%) of those developed TD. Health characteristics and some preventive attitudes are reported in table 2.

**Table 2:** Health characteristics and TD-preventive attitudes abroad (N = 2800).

	**Total**^**1)**^	subgroups			***p *value**^**2)**^
		TD n = 962	mild diarrhoea n = 303	no diarrhoea abroad n = 1535	
Body Mass Index	n = 2789				0.734
<18.5	91 (3.3)	32 (35.2)	9 (9.9)	50 (54.9)	
18.5-29.9	2612 (93.7)	892 (34.2)	283 (10.8)	1437 (55.0)	
≥30.0	86 (3.0)	34 (39.5)	9 (10.5)	43 (50.0)	
Allergy (MD assessed) ^3)^	n = 2785				0.721
none	1837 (66.0)	627 (34.1)	205 (11.2)	1005 (54.7)	
any	632 (34.0)	224 (35.4)	59 (9.3)	349 (55.2)	
allergic asthma	110 (17.5)	50 (45.5)	11 (10.0)	49 (44.5)	
allergic asthma vs. others					0.013
Comedication^3)^	n = 2800				0.741
none	2475 (88.4)	853 (34.5)	265 (10.7)	1357 (54.8)	
any	325 (11.6)	109 (33.5)	38 (11.7)	178 (54.8)	
mental/behavioural ^4)^	52 (16.8)	25 (23.6)	5 (9.6)	22 (13.3)	
mental/behavioural vs. others					0.031
History of diarrhoea	n = 1843	n = 674	n = 215	n = 954	0.009
none	855 (46.4)	282 (33.0)	99 (11.6)	474 (55.4)	
TD ever experienced	955 (51.8)	377 (39.5)	110 (11.5)	468 (49.0)	
Don't remember	33 (1.8)	15 (45.5)	6 (18.2)	12 (36.4)	
History of TD incapacitation	n = 1029	n = 373	n = 120	n = 497	0.139
low	528 (51.3)	197 (37.3)	65 (12.3)	266 (50.4)	
intermediate to severe	501 (48.7)	215 (42.9)	55 (11.0)	231 (46.1)	
Subjective diarrhoea susceptibility	n = 1656	n = 584	n = 186	n = 886	0.001
low	617 (37.3)	186 (30.1)	54 (8.7)	377 (61.1)	
intermediate	974 (58.8)	363 (37.3)	125 (12.8)	486 (49.9)	
high	65 (3.9)	35 (53.8)	7 (10.7)	23 (35.4)	
Diarrhoea episode <4 months before index travel^5)^	n = 2800				<0.001
	347 (12.4)	172 (49.6)	42 (13.9)	133 (38.3)	
Catering^3)^	n = 2323	n = 792	n = 252	n = 1279	
Buffet meals	2190 (94.3)	742 (33.9)	234 (10.7)	1214 (55.4)	0.380
Private/family meals	982 (42.3)	374 (38.1)	91 (9.3)	517 (52.6)	0.001
Street vendors meals	882 (38.0)	310 (35.1)	98 (11.1)	474 (53.7)	0.389
Adherence to *"cook it, boil it, peel it or forget it"*	n = 2318				0.299
	1428 (61.6)	477 (33.4)	160 (11.2)	791 (55.4)	

**NOTE**. *P *< 0.05 was considered statistically significant. NS, not significant. MD is medical doctor, TD travellers' diarrhoea.

^1) ^Included mentioned subgroups and mild diarrhoea cases

^2) ^chi square test. TD patients were compared to those with no diarrhoea abroad.

^3) ^Multiple answers possible

^4) ^Mainly antidepressive medication (n = 49)

^5) ^Pre-existing FGID (Functional Gastrointestinal Disease) patients and subjects with diarrhoea unresolved or lasting ≥14 d within this diarrhoea episode <4 months before index travel were excluded as defined in the exclusion criteria

### Factors influencing TD

Table 3 shows risk factors independently associated with TD with increasing age being the only protective one. We found no significant associations, either with other reported allergies (e.g. hay fever, atopic dermatitis), nor with other pre-travel co-medications (e.g. hormonal). Seasonality and gender were not associated to TD rates.

**Table 3:** Odds ratios (OR) for the risk factors of developing TD.

Variable	Univariate OR N = 2800	Multiple OR N = 2565	Final model OR N = 2565
Gender^1)^	0.90 (0.77-1.05)	1.01 (0.80-1.28)	0.96 (0.80-1.15)
Age (years)^2) 3)^	0.98 (0.98-0.99)	0.98 (0.97-1.00)	0.98 (0.98-0.99)
Weeks of stay^3)^	1.26 (1.20-1.33)	1.27 (1.17-1.37)	1.28 (1.21-1.35)
Visiting Friends and Relatives^2)^	1.62 (1.06-2.48)	1.41 (0.85-2.33)	
Smoking^1)^	1.11 (0.94-1.32)	1.06 (0.82-1.36)	
Daily alcohol drinking^1)^	0.94 (0.79-1.17)	1.01 (0.76-1.34)	
Allergic asthma^1)^	1.62 (1.11-2.38)	1.70 (0.95-3.07)	1.67 (1.10-2.54)
Psychiatric co-medication^1)^	1.79 (1.05-3.08)	1.88 (0.94-3.75)	2.11 (1.17-3.80)
Diarrhoea pre-travel^1)^	2.07 (1.65-2.59)	1.78 (1.31-2.43)	2.03 (1.59-2.54)
TD independent fever^1) 4)^	8.64 (4.01-17.85)	5.72 (2.27-14.38)	6.56 (3.06-14.04)
Malaria chemoprophylaxis^1)^	1.19 (0.98-1.44)	1.31 (1.00-1.72)	1.38 (1.12-1.70)
Consuming tap water abroad^1)^	1.08 (0.82-1.41)	0.88 (0.62-1.25)	
Self-reported adherence to *"cook it, boil it, peel it or forget it" *^1)^	0.91 (0.76-1.09)	1.00 (0.79-1.27)	
Body Mass Index^3)^	1.01 (0.99-1.04)	1.05 (1.01-1.09)	1.04 (1.01-1.08)
Adverse life event pre-travel^1)^	0.94 (0.72-1.22)	0.98 (0.66-1.46)	
High stress level pre-travel^1)^	1.11 (0.84-1.47)	0.94 (0.62-1.42)	

**NOTE**. OR: Odds ratio

^1) ^Reference group of variable: all others. For Gender Male. For Visiting Friends and Relatives Tourists and Business Travellers, for Smoking Non-smokers (previous smokers included), for Daily alcohol drinking No daily alcohol drinking reported, for Allergic asthma No allergic asthma reported, for Psychiatric co-medication No Psychiatric co-medication reported, for Diarrhoea pre-travel No diarrhoea pre-travel, for TD independent fever All others, for Malaria chemoprophylaxis No malaria chemoprophylaxis reported, for Consuming tap water abroad No such consuming reported, for Self-reported adherence to "*cook it, boil it, peel it or forget it*" Self-reported non-adherence, for Adverse life event No adverse life event reported pre-travel, for High stress level Very low to intermediate stress level pre-travel.

^2) ^Some missing data which accounts for ~10% less of N in the Final model.

^3) ^Continuous variables

^4) ^Imputation of missing data for later inserted question using the pattern of gender, destination continent and education variables.

## Discussion

In view of the fact that the TD incidence has been reduced in some countries such as Jamaica [16] and Thailand [17], updated worldwide regional incidence data were long overdue. Overall, we observe a substantial decrease in the two weeks incidence rate, nowhere did we find rates exceeding 50 or even 60% as documented in a large study conducted a decade ago (3, 4). Most regions which were high risk remained in high risk destination group [2,3,8,18,19]. Particularly East Asia, including China, Southeast Asia and to a lesser degree Latin America, showed a marked decrease in classic TD risk. Known high-risk TD destinations such as the Middle East, North Africa and the Caribbean have been underrepresented in our study, since visitors to those destinations generally do not consider a pre-travel consultation as indicated [20]. Some of these areas however, recently experienced a similar decrease in fecal-orally transmitted diseases[21]. For TD we confirmed younger age and longer travel duration as risk factors. In contrast, neither smoking nor consuming alcohol nor gender significantly influenced the occurrence of TD. TD patients, who had previously experienced TD, reported a significantly higher susceptibility to diarrhoea (*p *= 0.0127); that may be associated with genetic factors [22,23].

Allergic asthma, mental/behavioural co-medication, a high BMI, and a TD-independent fever episode to our knowledge have so far never been considered risk factors for TD. Such findings, however, may be clinically relevant and enhanced preventive measures to protect travellers vulnerable to multiple health impairments abroad should be considered. Allergic asthma as a risk factor needs further exploration [24,25], however both, atopic dermatitis and hay fever did not influence TD in our cohort. Within psychiatric co-medication antidepressants might be associated with diarrhoea as a side effect [26,27]. An elevated BMI resulted in a marginally increased risk ratio, similarly to being a predisposing factor for community-based diarrhoea [28-30]. Obese people have a higher food intake, and may thus consume a larger inoculum of pathogens. Lastly, a fever episode independent of TD was suggestive of some low-grade inflammatory and/or immunological processes [31,32]. Our questionnaire did not allow to assess the chronology of TD independent fever.

Loperamide was widely used to treat TD in our cohort, which is consistent with other reports [10,33] and some of the guidelines [34,35]. Only a minority of 6.8% of all cases relied on an antibiotic therapy for TD, which in most cases was carried in their travel medical kit. Almost 10% turned to a local pharmacy, potentially exposing themselves to the risk of fake medication abroad or receiving medication stored at inadequately temperatures [36,37].

Our study design with inherent selection bias [38] and missing chronology of TD treatment did not allow evaluation of the various treatment schedules. However, patients without antibiotic TD treatment (most using loperamide) were found to have significantly more TD episodes; targeted studies are needed to determine whether loperamide is associated with recurrent TD episodes.

Half of our study population had a university degree, travelling mostly as tourists but also for business. They might spend more money on travelling and be more likely to ask for pre-travel health advice. Hence, our results, although not ideal with respect to behaviour, may reflect a rather high socio-economic stratum. As our study participants all obtained the usual advice on hygiene measures to prevent TD, and as many were non compliant these approaches need to be improved, or other means of prophylaxis offered. For instance, an alternative approach may be to increase the public and tourism industry demand for food safety to reduce the burden of TD among travellers [39]. Previous studies often questioned travellers at the airport before or during the flight home, this one, however, some days after return. Thus, this latter study would also include TD which occurred in those first days back home, and one would expect slightly higher rates as compared to the older studies.

## Conclusion

This study shows that diarrhoea remains a very frequent health problem in travellers, although there is a trend to reduced incidence rates in various parts of the world. Besides well-known risk factors like duration of stay, young adult age, etc., also a patients' history of allergic asthma, of pre-travel diarrhoea and of TD-independent fever will need further investigation.

## Competing interests

RS has accepted fees or reimbursement for actively participating in education, consulting, advisory boards, research and also for attending meetings from Crucell, DrFalk Pharma, Intercell, Novartis, Salix Pharmaceuticals, and Santarus. All other authors report no conflict of interest.

## Authors' contributions

MM and RS were involved in the study design and supervision. RP and MM were involved in data collection. RP, MM and RS were involved in the analysis and interpretation of the data. AT provided statistical expertise. All authors drafted the article and were responsible for critical revision for intellectual content. All authors gave final approval to the manuscript.

## Pre-publication history

The pre-publication history for this paper can be accessed here:

http://www.biomedcentral.com/1471-2334/10/231/prepub
